# Patients’ Perspectives on Type 2 Diabetes, Vitamin B12 Deficiency, and Supplementation in Buraidah, Saudi Arabia

**DOI:** 10.7759/cureus.55345

**Published:** 2024-03-01

**Authors:** Osama O Alomar, Unaib Rabbani

**Affiliations:** 1 Family Medicine, Family Medicine Academy, Qassim Health Cluster, Buraidah, SAU

**Keywords:** vitamin b12, supplementation, saudi arabia, metformin, diabetes

## Abstract

Objectives

Long-term metformin is associated with vitamin B12 deficiency. There is a significant misunderstanding among both physicians and patients regarding vitamin B12 indications. This study aims to identify knowledge gaps and attitudes toward vitamin B12 among diabetic patients.

Materials and methods

A cross-sectional study was conducted among type 2 diabetic patients visiting four primary healthcare centers in Buraidah, Saudi Arabia. The data were collected using a structured self-administered questionnaire. Data were collected on diabetes and vitamin B12 knowledge and attitude toward vitamin B12 supplementation. Logistic regression analyses were used to assess the predictors of knowledge and attitude.

Results

Of the 388 participants, 192 (49.5%) were male. About 84.5% of the total diabetics were on metformin. Nearly three-fourths (72.7%) were taking vitamin B12. A large proportion, 160 (41.2%), believed that it is necessary to take vitamin B12 for every diabetic patient. Nearly half, 193 (49.7%), did not know the factors affecting vitamin B12 deficiency. Young (less than 39 years) diabetics were more likely to perceive that vitamin B12 is recommended for all diabetics, OR (95% CI) [6.26 (1.86-21.06)], as compared to participants aged more than 59. Similarly, younger patients were more likely to assume vitamin B12 necessary, OR (95% CI) [3.71 (1.26-10.89)].

Conclusion

We found the knowledge and attitude of diabetic patients regarding vitamin B12 to be poor. It is, therefore, recommended that primary health care providers educate their patients about vitamin B12 supplementation to reduce the number of medications and financial burden. Further large-scale studies are also needed to generate stronger evidence of the problem.

## Introduction

Diabetes mellitus (DM) type 2 is a chronic disease characterized by glucose intolerance and hyperglycemia. These patients have decreased levels of insulin, resistance to insulin action, or both. This results in prolonged exposure to hyperglycemia and variability in glucose levels, which causes acute and chronic complications that are divided into microvascular and macrovascular complications. It is classified into different types based on its etiology [1].

Type 2 diabetes is one of the most prevalent noncommunicable diseases worldwide. According to the WHO, the number of diabetic patients increased from 108 million in 1989 to 422 million in 2014 [2]. Locally, Saudi Arabia also has a high prevalence of DM (32.8% of the population [P1]) according to a systematic review. The researcher also predicted that it will reach 45.36% in 2030 [3].

Considering the high incidence and prevalence of diabetes and the expected numbers for the future, the physical and financial burden on the patients and the healthcare system will increase as the diabetic patient, on average, spends about 2.3 times more than those without DM [4]. In Saudi Arabia, diabetic patients spend almost $3,686 [P2] on medical expenses compared with $380 without DM [5].

Type 2 diabetes management is divided into non-pharmacological measures, such as diet and exercise, and pharmacological treatment, subdivided into insulin-dependent and noninsulin-dependent. Metformin is the initial pharmacological treatment for type 2 diabetes. It has been shown to reduce hepatic glucose production, but the exact mechanism of action is not fully understood [6]. Although metformin is a simple and inexpensive medication, many patients experience gastrointestinal side effects in the short term and vitamin B12 deficiency in the long term [7].

The vitamin B12 deficiency caused by metformin depends on many factors, among which dose and duration are predominant. It has been estimated that for every 1 g/day of metformin, the risk of developing vitamin B12 deficiency increases by 2.88% (95% confidence interval [CI], 2.15-3.87) [8]. Among those using metformin for more than three years, the adjusted odds ratio (OR) was 2.39 (95% CI, 1.46-3.91) compared to those taking metformin for less than three years [9]. A case-control study found that 22.5% of type 2 diabetics on metformin (compared with 7.4% of the control group) have vitamin B12 deficiency even after excluding proton pump inhibitors and histamine H2 antagonists 17.9% versus 5.6% [P3] [10].

There is an association between metformin use and vitamin B12 deficiency, and clear guidelines exist regarding vitamin B12 supplementation [11]. However, evidence suggests that there are misunderstandings among physicians. A study from Saudi Arabia concluded that 72.2% of primary care physicians would test vitamin B12 [P4] levels for type 2 diabetics taking metformin, and 34.8% would test them annually. Pain, tingling sensation, burning sensation, loss of protective sensation, and numbness are the five symptoms of early DM neuropathy. However, 45.5% of physicians would not treat DM neuropathy with vitamin B12. Fifty-seven percent would test vitamin B12 levels in diabetic patients taking metformin. About 70% of them prescribe vitamin B12 supplements to treat DM neuropathy [12]. These findings indicate variations in the knowledge and practices of primary care physicians with respect to B12 deficiency and supplementation among diabetic patients on metformin. Such variations among the physicians are expected to affect the patients' knowledge and attitudes as well.

No published guideline recommends routine screening for vitamin B12 among diabetic patients. Still, it is acceptable to screen before starting metformin to have a baseline level and screen annually in the elderly with a history of long-term use of metformin in high doses (more than three to four years, more than 2 g/day) [13]. Regardless of the etiology, vitamin B12 supplementation should be 1000 μg daily for one week and then a weekly dose for four weeks [13].

Generally, we note that many patients are convinced that it is necessary to have vitamin B12 supplements as long as they are on metformin without knowing its medical purpose or clear indications for its use. As we mentioned before, many physicians have a role in this misunderstanding by not clarifying the indication for and duration of the intervention.

This study aimed to find the gaps in diabetic patients’ knowledge and attitudes about vitamin B12 deficiency and supplementation. This information will be important for primary healthcare providers in planning the treatment and education of diabetic patients. This will help decrease the burden on patients and health systems by preventing unnecessary medication.

## Materials and methods

Methods

A cross-sectional study was conducted in four selected primary healthcare (PHC) centers in Buraidah. Buraidah is the capital and largest city of the Qassim region, with an estimated population of 600,000. This study was conducted among adult type 2 diabetic patients across all age groups attending PHCs in Buraidah.

Sample size calculation

The sample size was determined using the WHO manual “Sample Size Determination in Health Studies.” We assumed an expected proportion of 50% of type 2 diabetic patients having correct knowledge and attitudes toward vitamin B12 supplementation as no similar studies were available. Using a proportion of 50% would give us a maximum sample size. At a 95% CI and 5% margin of error, the required sample size was 385 diabetic patients.

Sampling strategy

We included four PHC centers located north, south, east, and west sectors of Buraidah. The PHC centers were selected based on the catchment population. Within each sector, the PHC center with the largest catchment population was selected. The required sample was divided equally among four PHCs; therefore, 100 diabetic patients were required from each of the centers. Within the center, the participants were selected consecutively through convenient sampling till the required number was achieved. All the type 2 diabetic patients who met our eligibility criteria were invited to participate in the study.

Inclusion and exclusion criteria

All adult diabetic type 2 patients of either gender visiting the selected healthcare centers were eligible to participate. We excluded patients with intellectual disabilities.

Data collection tool

Data were collected using a structured self-administered questionnaire. It included age, gender, date of diabetes diagnosis, duration of metformin use, metformin dose, vitamin B12 levels, if available, previous diagnosis of diabetic neuropathy, and general knowledge about DM, metformin, and diabetic neuropathy.

Data collection procedure

The questionnaire was distributed directly to the patients by undergraduate medical students trained in collecting data. Participants were informed about the study’s purpose and procedure. After assessing potential participants for eligibility, informed consent was obtained from those who met the eligibility criteria. Data collectors were present to provide any clarification if the need arise.

Data analysis

The data was analyzed using the Statistical Package for Social Sciences (SPSS Statistics for Windows, version 21.0; IBM Corp., Armonk, NY). Descriptive analysis measured the frequencies and proportions of categorical variables, while mean with standard deviations was calculated for continuous variables. Multivariable logistic regression analyses were used to find the factors associated with perceived indication and necessity of vitamin B12 supplementation. The OR and the associated 95% CI were calculated.

Ethical considerations

Ethical approval was given by the Qassim Regional Bioethics Committee (607/44/7572, dated December 12, 2022). Informed consent was obtained from all participants, and confidentiality was maintained as the names and IDs of participants were not collected.

## Results

The total participants in the research were 388 patients, 13.6% of participants were below 39 years, and 41.5% of them were above 60 years. Half of the participants were males (49.5%). The majority of the participants were married (90.8%), and 94.1% were Saudi nationals (Table 1).

**Table 1:** Sociodemographic characteristics of participants (n = 388)

Variables	% (n)
*Age (years)*	
Less than 39	13.6 (53)
Between 40-59	44.4 (174)
More than 60	41.5 (161)
*Gender*	
Male	49.5 (192)
Female	50.5 (196)
*Marital status*	
Single	9.3 (36)
Married	90.8 (352)
*Nationality*	
Saudi	94.1 (365)
Non-Saudi	5.9 (23)

The findings showed that nearly half (48.7%) of the participants were diagnosed with diabetes for more than 10 years. About 84.5% of the total diabetics were on metformin, but they were different in terms of the number of years and the dose that they had been using. The findings also showed that 72.7% were taking vitamin B12, and 55.2% of the total participants were taking it for more than a year (Table 2).

**Table 2:** History of the participants regarding DM and vitamin B12 DM: Diabetes mellitus.

Variables	% (n)
*How long you have been diagnosed with diabetes?*	
Less than 5 years	30.6 (119)
5-10 years	20.6 (80)
More than 10 years	48.7 (189)
*Are you on metformin?*	
Yes	84.5 (328)
No	15.5 (60)
*If yes, how long have you been using it?*	
Less than a year	7.7 (30)
1-5 years	22.4 (87)
5-10 years	19.1 (74)
More than 10 years	35.3 (137)
Not on metformin	15.5 (60)
*What dose of metformin are you on (yes)?*	
500 mg	26.8 (104)
1 g	24.2 (94)
1.5 g	29.4 (114)
2 g or more	4.1 (16)
Not on metformin	15.5 (60)
*Do you take vitamin B12?*	
Yes	72.7 (282)
No	27.3 (106)
*How long have you been taking vitamin B12?*	
Less than a month	3.1 (12)
1-6 months	8.8 (34)
6-12 months	5.7 (22)
More than a year	55.2 (214)
Not on vitamin B12	27.3 (106)

Table 3 presents the knowledge and attitude of diabetic patients regarding DM. Commonly reported risk factors for developing DM were hereditary (65.5%) and obesity (54.6%). Frequent urination (78.9%) and increased thirst (64.9%) were one of the commonly reported early signs of diabetes. Date also showed that they correctly believe that diabetes affects the heart (68.3%), the kidneys (76.5%), the eyes (87.6%), and the nerves (80.7%). About 73% answered yes to the relationship between high blood pressure and worsened diabetic outcomes (Table 3).

**Table 3:** Knowledge and attitude of diabetic patients regarding DM DM: Diabetes mellitus.

Variables	% (n)
*Risk factors of DM (Yes)*	
Hereditary	65.5 (254)
Obesity	54.6 (212)
Smoking	18 (70)
High sugar diet	49 (190)
Don’t know	17 (66)
*Early symptoms of diabetes (yes)*	
Frequent urination	78.9 (306)
Increased thirst	64.9 (252)
Increased hunger	30.2 (117)
Weight loss	35.8 (139)
Don't know	14.4 (56)
Do you think diabetes affects the heart?	
Yes	68.3 (265)
No	7.7 (30)
Don't know	24 (93)
*Do you think diabetes affects the kidneys?*	
Yes	76.5 (297)
No	6.2 (24)
Don't know	17.3 (67)
*Do you think diabetes affects the eyes?*	
Yes	87.6 (370)
No	2.1 (8)
Don't know	10.3 (40)
*Do you think diabetes affects the nerves?*	
Yes	80.7 (313)
No	4.6 (18)
Don't know	14.7 (57)
*Should a diabetic patient check his/her blood sugar?*	
Yes	85.6 (332)
No	7.2 (28)
Don't know	7.2 (28)
*Do you think regular exercise helps in glucose control?*	
Yes	92.3 (358)
No	1.5 (6)
Don't know	6.2 (24)
*Do you think that in a diabetic patient, high blood pressure can worsen diabetes?*	
Yes	72.9 (283)
No	7.2 (28)
Don't know	19.8 (77)

Knowledge and attitude regarding vitamin B12 deficiency and supplementation are presented in Table 4. It was found that most of the questions were answered incorrectly by a large number of the participants. A large proportion (41.2%) believed that it is necessary to take vitamin B12 for every diabetic patient. Similarly, a majority (70%) thought routine testing for vitamin B12 was necessary. Less than half (43.6%) of the participants agreed that vitamin B12 deficiency is related to metformin, but the majority (49.7%) did not know the factors affecting it (Table 4).

**Table 4:** Knowledge and attitude of diabetic patients regarding vitamin B12 deficiency and supplementation

Variables	% (n)
*Vitamin B12 supplementation is necessary for:*	
Every diabetic patient	41.2 (160)
Patients who are taking metformin	34 (132)
Patients who have peripheral neuropathy symptoms	11.9 (46)
None of the above	5.4 (21)
Don't know	7.5 (29)
*Do you think that routine testing for vitamin b12 is necessary?*	
Yes	70.1 (272)
No	8.8 (34)
I don’t know	21.1 (82)
*If yes, how frequently?*	
Every 6 months	39.7 (154)
Every 1 year	20.4 (79)
Every 5 years	6.7 (26)
For symptomatic patients only	0.5 (2)
No need	3.1 (12)
Don't know	29.6 (115)
*Do you think that vitamin B12 is a treatment for diabetic peripheral neuropathy?*	
Yes	61.6 (239)
No	17 (66)
I don’t know	21.4 (83)
*Early signs of diabetic peripheral neuropathy (DPN) (more than one can be selected)*	
Pain	55.9 (217)
Burning sensation	47.4 (184)
Tangling sensation	40.2 (156)
Numbness	55.9 (217)
Loss of protective sensation (LOPS)	18 (70)
Don't know	15.7 (61)
*Do you think that there is a relationship between metformin and vitamin B12 deficiency?*	
Yes	43.6 (169)
No	16.5 (64)
I don’t know	39.9 (155)
*If yes, what are the factors that affect it? (More than one can be selected)*	
Longer metformin duration	16 (62)
High metformin dosage	5.2 (20)
Associated with dose and duration	11.9 (46)
Irrelevant to dose or duration	17.3 (67)
I don’t know	49.7 (193)
*Do you think that every diabetic patient who is on metformin should be on vitamin B12 supplementation?*	
Yes	69.6 (270)
No	13.4 (52)
I don’t know	17 (66)

The multivariable regression analysis shows that the perceived indication of vitamin B12 was more than six times higher among less than 39-year-old participants, adjusted OR (95% CI) [6.26 (1.86-21.06)], as compared to participants aged more than 59. On the other hand, males were less likely to perceive vitamin B12 indication, adjusted OR (95% CI) [0.41 (0.19-0.90)] as compared to females. Those having a shorter duration of DM were more likely to indicate vitamin B12 as compared to those who had DM for more than 10 years, 5-10 years adjusted OR (95% CI) [3.20 (1.04-9.85)], and less than five years, adjusted OR (95% CI) [4.80 (1.72-13.36)]. We did not find any association between vitamin B12 indication with respect to marital status and current vitamin B12 supplementation.

The data showed that compared to age above 59, younger patients less than 39 years were more likely to assume vitamin B12 to be necessary, adjusted OR (95% CI) [3.71 (1.26-10.89)]. There was no association of necessity assumption with gender, duration of DM, and marital status. When we compared their idea of indication with taking vitamin B12, we found that those who are currently taking vitamin B12 are less likely, adjusted OR (95% CI) [0.22 (0.11-0.45)], to report vitamin B12 necessity as compared to those who are not taking vitamin B12 (Table 5).

**Table 5:** Factors associated with perceived indication and necessity of vitamin B12 OR: Odds ratio; CI: Confidence interval.

Variable	Indication of vitamin B12		Necessity of vitamin B12	
	Adjusted OR (95% CI)	p-value	Adjusted OR (95% CI)	p-value
*Age (years)*				
Less than 39	6.26 (1.86–21.06)	0.003	3.71 (1.26–10.89)	0.017
40–59	1.40 (0.49–0.98)	0.521	1.03 (0.42–2.51)	0.938
More than 59	1		1	
*Gender*				
Male	0.41 (0.19–0.90)	0.026	1.28 (0.63–2.57)	0.489
Female	1		1	
*DM duration*				
Less than 5 years	4.80 (1.72-13.36)	0.003	1.86 (0.79–4.36)	0.152
5-10 years	3.20 (1.04-9.85)	0.042	1.36 (0.50–3.65)	0.538
More than 10 years	1		1	
*Marital status*				
Single	1.88 (0.68–5.16)	0.217	1.77 (0.66–4.72)	0.254
Ever married	1			
*Do you take vitamin B12?*				
Yes	0.56 (0.26–1.18)	0.132	0.22 (0.11–0.45)	<0.001
No	1		1	

## Discussion

This study aims to find the knowledge gaps among type 2 diabetic patients regarding vitamin B12 deficiency and supplementation. The need for this research comes from practice in the clinic as many patients insist on beginning vitamin B12 supplements or are already on it. We found that patients of younger age are more likely to think it is indicated and necessary to take vitamin B12. We also discovered that females are more likely to believe that it is indicated and necessary to take vitamin B12.

Knowledge about DM is essential in controlling the disease and preventing complications. In our study, participants showed a higher level of knowledge about DM risk factors. About 79% chose frequent urination, and 65% chose increased thirst as early symptom of DM. Similarly, a 2022 study done in Hail, Saudi Arabia, showed that frequent urination (94.6%) and increased thirst (86.6%) were the commonly reported DM symptoms [14]. In the same study, they found that participants were aware that the most prominent risk factor for DM is a family history (93.1%), with being overweight or obese following closely behind (89.7%) [14]. In our study, participants chose hereditary 65% and obesity 55% as the most common DM risk factors.

Another important aspect related to knowledge of DM is understanding vitamin B12 deficiency and supplementation and the relation between DM, metformin, and vitamin B12. Our study data showed that 41% of participants think that every person with DM should take vitamin B12 supplementation without apparent justification for that position. About one-third (34%) perceived that patients taking metformin should take the supplement. Nevertheless, almost 50% did not know the relation between metformin and vitamin B12 deficiency. In our study, about 61% of the participants believed that vitamin B12 deficiency is the same as diabetic peripheral neuropathy. However, these two are different entities in reality. On the other hand, we also observed that only a small proportion of the participants reported diabetic peripheral neuropathy as one of the symptoms. This indicates that there is a large gap in the knowledge of patients as they were unclear about the difference between diabetic peripheral neuropathy and vitamin B12 deficiency. This finding corroborates a study among primary care physicians in the same setting, which showed poor understanding of vitamin B12 supplementation and diabetic peripheral neuropathy [12]. This is an important finding and has implications for practitioners in primary care to properly educate diabetic patients about diabetic complications and the side effects of medications used in DM management.

Despite the established connection between metformin usage and vitamin B12 deficiency, along with clear guidelines regarding vitamin B12 supplementation, patients appear to lack understanding. Similar was the situation for the physicians as a local study conducted in Saudi Arabia indicated that 70.6% of physicians would provide vitamin B12 supplements as a treatment for diabetic neuropathy [12].

However, vitamin B12 deficiency commonly occurs in around 6% of elderly patients aged ≥60 years, with its prevalence increasing with age [15]. We found that younger people with DM were more likely to assume that it is indicated and necessary for them to take vitamin B12 compared to older patients. We assume that the reason for that is younger patients tend to be stricter and more careful regarding their health. Another possible reason for this finding could be the lack of knowledge about the disease, medications, and complications. Conversely, another study showed that one of the poor compliance factors is young age [16]. It is therefore necessary to explore the reasons behind such responses by the younger diabetics through in-depth investigations.

Furthermore, clinicians managing younger DM patients should also provide adequate education to their patients to avoid any misconceptions. The data also revealed that females are more likely to indicate a need for vitamin B12 than males. This is supported by a study conducted in Libya in 2017 [16], which showed females have a significantly lower level of vitamin B12 than males. One previous study revealed an inverse relationship between body mass index (BMI) and vitamin B12 level [17]. Generally, females tend to have significantly higher BMI than males [18]. Therefore, females may have more pronounced symptoms of B12 deficiency and thus assume B12 supplementation is a necessary component of DM management. This finding indicates that younger female diabetic patients need special attention from care providers in terms of educating them about vitamin B12 supplementation as part of DM management. There is also a need for further research to explore whether the females’ specific attitudes toward B12 supplementation are due to their physiology or due to other sociocultural factors.

To the best of our knowledge, this study is one of its kind to assess the diabetic patient's knowledge and attitude about vitamin B12 supplementation. We used previously validated questionnaires for the measurements. Furthermore, to capture population variation in terms of sociodemographics, we recruited participants from PHCs located in different geographical locations within Buraidah.

However, certain limitations need consideration while interpreting the results of this study. First, this study was done in four different PHCs in four parts of Buraidah city, which limits its generalizability to the Saudi diabetic population. Another limitation is self-reported knowledge and attitudes, which may be subject to bias. However, we assume this to have a limited impact on the validity of results as data were collected in privacy and anonymously.

## Conclusions

This study found deficiencies in the knowledge and attitude toward vitamin B12 among type 2 diabetes patients. We also found differences in the knowledge and attitude with respect to age and sex. It is, therefore, recommended that PHC providers must educate their patients about vitamin B12 supplementation. This may help reduce the number of medications and the financial burden of unnecessary medications. Further large-scale studies are also needed to generate more robust evidence of the problem.

## Appendices

**Table 6:** Questionnaire

Part I: Personal data
· Age: …………………………………
· Gender: ☐ Male ☐ Female
· Marital status: ☐ Single ☐ Married ☐ Divorced/separated
· Nationality: ☐ Saudi ☐ Non-Saudi
· How long you have been diagnosed with diabetes: ………………………..
· Are you on Metformin: ☐ Yes ☐ No
· If yes how long have you been using it: : ☐ Less than a year ☐ 1-5 years ☐ 5-10 years ☐ More than 10 years
· If yes what is the dose: ☐ 500 mg ☐ 1 gram ☐ 1.5 grams ☐ 2 grams or more
· Do you take vitamin B12: ☐ Yes ☐ No
· If yes how long: ☐ Less than a month ☐ 1-6 months ☐ 6-12 months ☐ More than a year
Part II: Knowledge and attitude regarding diabetes mellitus
1. What do you think is the major cause of diabetes? (more than one can be selected)
☐ Hereditary
☐ Obesity
☐ Smoking
☐ Eating too much sugar
☐ Don't know
2. What are the early symptoms of diabetes?
☐ Frequent urination
☐ Increased thirst
☐ Increased hunger
☐ Weight loss
☐ Don't know
3. Do you think diabetes affects the heart?
☐ Yes
☐ No
☐ Don't know
4. Do you think diabetes affects the kidneys?
☐ Yes
☐ No
☐ Don't know
5. Do you think diabetes affects the eyes?
☐ Yes
☐ No
☐ Don't know
6. Do you think diabetes affects the nerves?
☐ Yes
☐ No
☐ Don't know
7. Should a diabetic patient check his/her own blood sugar?
☐ Yes
☐ No
☐ Don't know
8. Do you think regular exercise helps in glucose control?
☐ Yes
☐ No
☐ Don't know
9. Do you think that in a diabetic patient, high blood pressure can worsen diabetes?
☐ Yes
☐ No
☐ Don't know
Part III: Knowledge and attitude regarding vitamin B12 deficiency and supplementation
1. Vitamin B12 supplementation is necessary for:
☐ Every diabetic patient
☐ Patients who are taking metformin
☐ Patients who have peripheral Neuropathy symptoms
☐ None of the above
2. Do you think that routine testing for vitamin b12 is necessary:
☐ Yes
☐ No
☐ I don’t know
3. If yes, how frequent:
☐ Every 6 months
☐ Every 1 year
☐ Every 5 years
☐ For symptomatic patients only
☐ No need
4. Do you think that vitamin B12 is the treatment for diabetic peripheral neuropathy (DPN)?
☐ Yes
☐ No
☐ I don’t know
5. Early signs of DPN (more than one can be selected)
☐ Pain
☐ Burning sensation
☐ Tangling sensation
☐ Numbness
☐ Loss of protective sensation (LOPS)
6. Do you think that there is a relation between metformin and vitamin B12 deficiency?
☐ Yes
☐ No
☐ I don’t know
7. Do you think that every diabetic patient who is on metformin should be on vitamin B12 supplementation?
☐ Yes
☐ No
☐ I don’t know
8. If yes, what are the factors that affect it? (more than one can be selected)
☐ Longer metformin duration
☐ High metformin dosage
☐ Associated with dose and duration
☐ Irrelevant to dose or duration
☐ I don’t know
